# Aspirin-Loaded Polymeric Films for Drug Delivery Systems: Comparison between Soaking and Supercritical CO_2_ Impregnation

**DOI:** 10.3390/pharmaceutics13060824

**Published:** 2021-06-02

**Authors:** Isabela Trindade Coutinho, Lígia Passos Maia-Obi, Mathilde Champeau

**Affiliations:** Center of Engineering, Modeling and Applied Social Sciences, Federal University of ABC, Santo Andre 09210-580, Brazil; isabela.coutinho@aluno.ufabc.edu.br (I.T.C.); ligia.maia@ufabc.edu.br (L.P.M.-O.)

**Keywords:** soaking, supercritical CO_2_, impregnation, drug-eluting implant, acetylsalicylic acid, controlled release, poly(l-lactic acid), polyethylene

## Abstract

Polymeric implants loaded with drugs can overcome the disadvantages of oral or injection drug administration and deliver the drug locally. Several methods can load drugs into polymers. Herein, soaking and supercritical CO_2_ (scCO_2_) impregnation methods were employed to load aspirin into poly(l-lactic acid) (PLLA) and linear low-density polyethylene (LLDPE). Higher drug loadings (DL) were achieved with scCO_2_ impregnation compared to soaking and in a shorter time (3.4 ± 0.8 vs. 1.3 ± 0.4% for PLLA; and 0.4 ± 0.5 vs. 0.6 ± 0.5% for LLDPE), due to the higher swelling capacity of CO_2_. The higher affinity of aspirin explained the higher DL in PLLA than in LLDPE. Residual solvent was detected in LLDPE prepared by soaking, but within the FDA concentration limits. The solvents used in both methods acted as plasticizers and increased PLLA crystallinity. PLLA impregnated with aspirin exhibited faster hydrolysis in vitro due to the catalytic effect of aspirin. Finally, PLLA impregnated by soaking showed a burst release because of aspirin crystals on the PLLA surface, and released 100% of aspirin within 60 days, whereas the PLLA prepared with scCO_2_ released 60% after 74 days by diffusion and PLLA erosion. Hence, the scCO_2_ impregnation method is adequate for higher aspirin loadings and prolonged drug release.

## 1. Introduction

Acetylsalicylic acid (aspirin) is a common anti-inflammatory drug and is widely prescribed as an anti-platelet to prevent cardiovascular events [1,2,3]. Although aspirin’s main administration route is oral, this route has poor bioavailability (40–50%) [3,4] and its prolonged use is associated with gastrointestinal mucosa ulcers and gastrointestinal hemorrhaging in severe cases [3,5]. In the literature, there is evidence that aspirin parenteral administration (non-oral) can reduce gastrointestinal side effects [6]. An alternative to oral administration is developing aspirin local delivery systems by loading the aspirin into polymeric implants for cardiovascular applications, such as stents [7,8,9,10], scaffolds [11,12,13], and gels [6].

Several strategies can be used to develop these polymeric drug delivery systems [14,15,16]. Soaking is a conventional method that consists of immersing the polymer in a drug-concentrated solution, allowing the drug to diffuse into its amorphous regions and be retained due to interactions with the polymeric chains [14,16,17]. In the final step, the solvent is removed, usually by evaporation [18,19]. The choice of solvent must be accurate: it must solubilize the drug and swell the polymer. Soaking is a simple method and can be used to load a variety of drugs according to the right combination of {solvent + polymer + drug}. In the literature, it has been used mainly to load drugs into sutures [18,19], scaffolds [20], and contact lenses [21,22,23,24].

The supercritical carbon dioxide (scCO_2_) impregnation is based on a similar principle as the soaking method, but in this case, scCO_2_ is the solvent. In its supercritical state, above the critical point of 31 °C and 7.4 MPa, CO_2_ has a high density and high diffusivity [25]. The high density provides scCO_2_ with a solvating power similar to liquids, solubilizing various nonpolar drugs with low molecular weight [26,27]. Moreover, its high diffusivity, similar to a gas, allows it to be absorbed into the amorphous regions of various amorphous and semicrystalline polymers [28,29]. Therefore, scCO_2_ can solubilize the drug and carry it into the polymeric matrix. Since CO_2_ is a gas at atmospheric conditions, the polymeric drug delivery system is obtained without solvent through a simple depressurization step, whereas the drug is trapped between the polymeric chains. Sutures [30,31], lenses [32,33], and textiles [34,35] have been loaded with drugs or other active pharmaceutical substances using this method.

Previous works have compared the soaking method and scCO_2_ impregnation method of drug delivery system development based on hydrogels and amorphous matrices [21,36,37]. To the best of our knowledge, such studies have not been performed on thermoplastic matrices, which are extensively used to develop biomedical implants. Moreover, the increased interest in developing drug-eluting implants and scaffolds of thermoplastics polymers using fused deposition modeling (FDM) 3D printing motivates the comparison of the impregnation methods, to decide which should be used to load the drugs into the filaments or printed device, depending on the desired release profile [20,38,39,40,41]. In order to select the most appropriate drug loading processes, it is necessary to understand its impact on drug loading, the polymer microstructure, the drug crystalline state, the in vitro polymer behavior, and the drug release profile.

In this paper, we compared the two methods of post-manufacture aspirin loading into polymeric matrices, to highlight their differences and verify if scCO_2_ impregnation is a suitable alternative to soaking. Poly(l-acid lactic) (PLLA) and linear low-density polyethylene (LLDPE) were chosen as model polymeric matrices due to their different physical-chemical properties and the fact that they can interact differently with aspirin and with the solvents. These two polymers are also widely used to produce implants [42,43,44,45]. The two impregnation methods were compared in terms of drug loading and the characteristics of each process (solvent absorption, residual solvent, impregnation time) for PLLA and LLDPE. Considering that PLLA resulted in higher drug loadings, it was selected for further characterizations. The impact of the impregnation method was evaluated on thermal properties, on the microstructure, in vitro degradation, and in vitro release.

## 2. Materials and Methods

### 2.1. Materials

**Figure 1:**
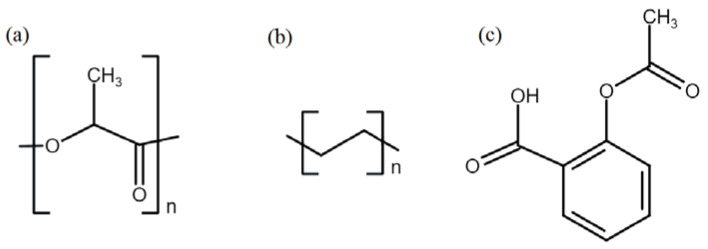
Chemical structures of (**a**) PLLA, (**b**) LLDPE, and (**c**) aspirin.

PLLA filament was purchased from Cliever (Belo Horizonte, MG, Brazil). LLDPE pellets were supplied by Braskem (São Paulo, SP, Brazil). Carbon dioxide (purity 99.985%) was purchased from Oxilumen (São Paulo, SP, Brazil). Acetylsalicylic Acid (aspirin) (purity 99.0%) was purchased from Sigma-Aldrich (Barueri, SP, Brazil) and was macerated before use to reduce particle size and facilitate its solubilization. The structures of the polymers and aspirin are presented in Figure 1. Silicone oil (Synth, 350 cps) was used for thermostated baths. Ethanol (purity 99%) and Isopropanol (purity 99%) were obtained from Synth (São Paulo, SP, Brazil) and used as received. Sodium chloride (NaCl), potassium chloride (KCl), sodium phosphate dibasic (Na_2_HPO_4_), monopotassium phosphate (KH_2_PO_4_), were purchased from Sigma-Aldrich and used as received to prepare the 1 M phosphate-buffered saline (PBS) solution for in vitro degradation and release analysis. Deuterated chloroform (CDCl_3_, 99.8%) containing 0.05% *v*/*v* tetramethylsilane (TMS) as the internal reference was purchased from Cambridge Isotope Laboratories and used in the NMR analysis.

### 2.2. Preparation of Polymeric Films

PLLA filaments cut in pieces of approximately 1 cm and LLDPE pellets as received were processed into films through hot pressing on a hydraulic press with heating (model SL-11, Solab, Piracicaba, Brazil). A pressure gradient of up to 6 tons was applied for 10 min at 215 °C to PLLA and at 165 °C to LLDPE, using Teflon sheets for the contact between the press and the samples. The films of 0.4 mm thickness were cut into squares of approximately 1 cm^2^, which corresponded to approximately 40 mg per sample.

### 2.3. Solvent Absorption and Residual Solvent

Ethanol and isopropanol were used as solvents for the soaking impregnation method, and CO_2_ was used as the solvent for the scCO_2_ impregnation method. The solvent absorption and residual solvent content in the polymeric matrices were investigated in the absence of aspirin and evaluated gravimetrically using a precision balance (10^−4^ g, model AY-220, Marte, Shimadzu, São Paulo, Brazil). The measurements were performed in triplicate. The residual solvent content of ethanol was also determined by proton nuclear magnetic resonance (1H NMR) analysis (Supplementary Materials Section S1.1).

In order to confirm the presence or absence of solvent, Fourier-transform infrared spectroscopy (FTIR) analyses were performed using a spectrophotometer (Perkin Elmer, Model Spectrum Two, São Paulo, Brazil) in attenuated total reflectance (ATR) mode (diamond doped with zinc selenide crystal). The absorbance spectra were obtained with 16 scans and a resolution of 2 cm^−1^, between 700 and 4000 cm^−1^, at room temperature.

#### 2.3.1. Ethanol and Isopropanol

Neat PLLA and LLDPE films were weighted (m_0_) and immersed in ethanol and isopropanol, respectively. The films were removed after 10 days of soaking to ensure maximal solvent absorption (Supplementary Materials Section S2, Figures S2 and S3). The surface was gently dried with tissue paper, and the films were weighed again (m_s_). The percentage of solvent absorption was calculated using Equation (1).

Solvent absorption = (m_s_ − m_0_)/m_0_ × 100 (1)

These samples were then dried at 80 °C for 3 h, for the solvent removal, and weighted (m_d_). The residual solvent was calculated using Equation (2):

Residual Solvent = (m_d_ − m_0_)/m_0_ × 100 (2)

#### 2.3.2. CO_2_

**Figure 2:**
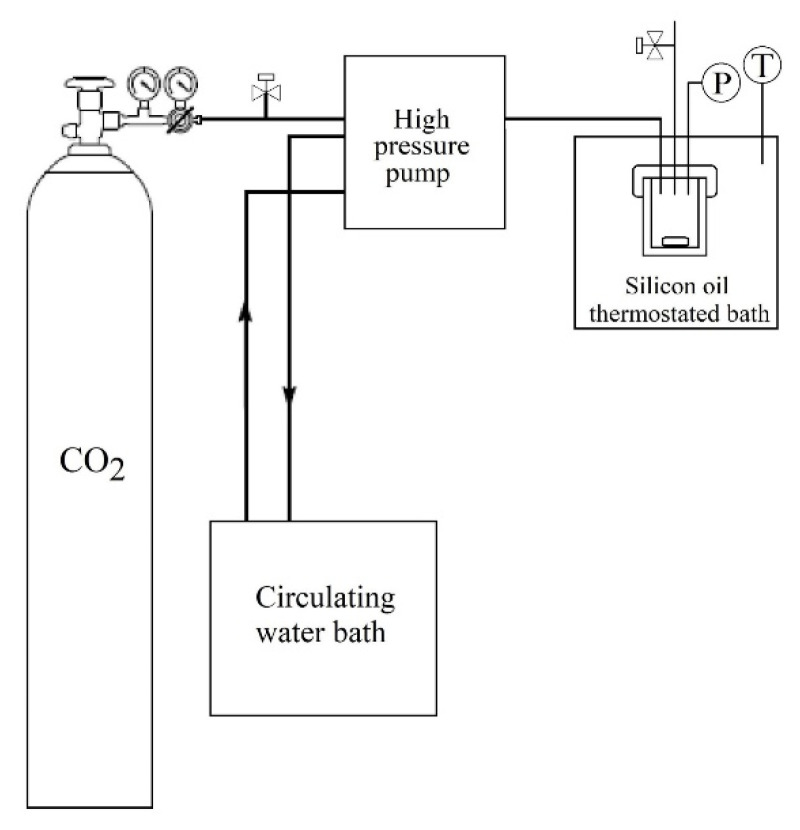
Schematic representation of the supercritical CO_2_ impregnation system.

PLLA and LLDPE films were weighted (m_0_) and placed vertically into a 10 mL stainless steel high-pressure cell. The reactor was immersed in a silicone oil thermostated bath at 80 °C, and CO_2_ was introduced into the reactor using a high-pressure pump (model SFC P-50A, Thar Technologies, Pittsburgh, PA, USA) until a pressure of 30 MPa was achieved (Figure 2). The temperature and pressure were kept constant for 3 h. The depressurization was performed by dipping the reactor in dry ice to freeze the CO_2_ to prevent its desorption from the polymeric matrices [46]. The samples were immediately weighed after being removed from the high-pressure cell (m_s_) and continuously weighted until mass stabilization (m_d_). The amount of CO_2_ (m_co2_) sorbed into the films during the scCO_2_ impregnation process was not desorbed during the depressurization process. Similar to the soaking process, the scCO_2_ absorption was calculated using Equation (1).

Preliminary measurements showed that the mass of the films was stabilized 5 days after their impregnation (m_d_). For this reason, all posterior analyses were performed after this period. Similar to the soaking method, the residual solvent was then obtained using Equation (2).

### 2.4. Impregnation

#### 2.4.1. Soaking Method

Five PLLA and LLDPE films were immersed in 100 mL of aspirin solution and kept at room temperature without stirring for 10 days. PLLA and LLDPE films were soaked in aspirin solution in ethanol (0.08 g∙mL^−1^) and isopropanol (0.1 g∙mL^−1^), respectively. After 10 days of soaking, the films were removed from the solutions, placed in a petri dish, and dried at 80 °C for 3 h in an oven for solvent removal.

#### 2.4.2. scCO_2_ Impregnation Method

The scCO_2_ impregnation was carried out in a batch process with the same protocol as previous work [47]. Three PLLA films and three LLDPE films were loaded into a 10 mL stainless steel high-pressure cell containing approximately 50 mg of aspirin, to ensure aspirin saturation throughout the experiment (2.8 mg∙mL^−1^), and a stirrer bar. The films were placed in the vertical position in a sample holder, ensuring the films did not touch each other and were physically separated from aspirin. As in the swelling solvent absorption measurements, the reactor was immersed in a silicone oil bath at 80 °C and the CO_2_ was introduced into the reactor until the pressure of 30 MPa was achieved. The temperature and pressure were kept constant for 3 h, and the magnetic stirring was maintained at 100 rpm. The depressurization was performed by dipping the reactor in dry ice (−78 °C) to freeze the CO_2_ and avoid aspirin desorption during venting [46]. The scCO_2_ conditions {80 °C; 30 MPa; 3 h} were chosen because they were above the glass transition temperatures (T_g_) of the PLLA (62 °C) and the LLDPE (−110 °C) and because they have been reported to achieve high aspirin loading in PLLA [46].

### 2.5. Drug Loading Quantification

The drug loading (DL%) was defined as the mass of aspirin impregnated per mass of polymer and was obtained using Equation (3):

DL% = (m_f_ − m_0_)/m_0_ × 100 (3)

where m_f_ is the film mass 5 days after impregnation for the scCO_2_ impregnation and the film mass after the drying step for the soaking method, and m_0_ is the film mass before the impregnation measured with precision balance (10^−4^ g, model AY-220, Marte, Shimadzu, São Paulo, Brazil). For each method, the impregnation was performed at least in triplicate, and the average drug loading is presented.

The difference between the drug loading of each polymer and method was statistically evaluated through ANOVA, using the Tukey test with 5% of significance.

### 2.6. Scanning Electron Microscope (SEM)

SEM analysis (JEOL JSM6010LA, São Paulo, Brazil) was used to evaluate possible aspirin precipitation on the surface of the films and morphological changes in the cross-section of PLLA samples. The fracture of the samples was performed by cryofracture using liquid nitrogen, and the samples were sputtered with a 15 nm layer of gold.

### 2.7. Thermal Analysis

The impact of the two impregnation processes on PLLA thermal properties and crystallinity was evaluated by differential scanning calorimetry (DSC) analyses (Q200, TA Instruments, Barueri, Brazil). The DSC curve of the neat LLDPE film was also obtained. The films were cut into small pieces, and 7 to 8.5 mg was sealed in aluminum pans. The samples were heated from 25 °C to 200 °C for PLLA and 150 °C for LLDPE with a heating rate of 10 °C∙min^−1^ (first heating), then cooled to −80 °C with a rate of 20 °C∙min^−1^ and heated up again to 200 °C for PLLA and 150 °C for LLDPE with a rate of 10 °C∙min^−1^ (second heating). The polymer crystallinity was obtained using Equation (4):

χ = (∆H_f_ − ∆H_c_)/(W × ∆H_100f_) × 100 (4)

where ∆H_f_ (J·g^−1^) is the experimental fusion enthalpy, ∆H_c_ (J·g^−1^) is the experimental crystallization enthalpy, W is the polymer mass fraction and ∆H_100f_ is the fusion enthalpy of 100% crystalline polymer, 93.1 J·g^−1^ for PLLA [48], and 288.7 J·g^−1^ for LLDPE [49].

### 2.8. X-ray Diffraction (XRD)

XRD analyses were performed to evaluate the crystalline state of impregnated aspirin in PLLA. The changes in PLLA crystallinity after the impregnation were also evaluated. The XRD patterns of the neat films, films only treated with solvent (prepared in Section 2.3), and impregnated films were obtained with a D8 Focus diffractometer (Bruker, Atibaia, Brazil), using Cu Kα_1_ radiation (wavelength = 1.54051 Å) at 40 kV and 40 mA, in the 2θ range of 5°–60° at the rate of 5°∙min^−1^. Before the analyses, the impregnated samples were gently cleaned with wet tissue paper to remove any precipitated aspirin from the surface.

### 2.9. In Vitro PLLA Degradation

The study of in vitro degradation was performed with neat PLLA, PLLA impregnated using the scCO_2_ method, and PLLA impregnated using the soaking method. PLLA was chosen because it has higher drug loadings and is known to degrade during its in vivo application, whereas LLDPE is expected to last longer (>years) [43,45]. Of each sample, 10 specimens were separately immersed in 30 mL of 1 M PBS, pH 7.4 at 37 ± 1 °C, following ASTM F1635-16 [50]. The pH of the PBS solutions was periodically monitored throughout the degradation study, and when a pH variation of ±0.2 was detected, the solution was replaced with fresh PBS. The PLLA films were removed at predetermined intervals and dried in a vacuum oven at 30 °C for 24 h. The degradation study was carried out for 112 days. ^1^H NMR analyses were performed to evaluate the decrease in the polymer’s number-average molecular weight (M_n_). Approximately 10 mg of sample was dissolved in 0.5 mL of CDCl_3_ and the spectra were recorded in a Varian VNMRS 500 MHz at 27 °C, using 800 transients. The number-average molecular weights for PLLA were estimated from the comparison of the signal integration of the PLLA methine ester end group (δ = 4.36 ppm) and the PLLA methine ester group (δ = 5.20 ppm) [51]. The PLLA crystallinity and thermal properties evolution were analyzed through DSC using the same protocol described in Section 2.8.

### 2.10. In Vitro Aspirin Release

The in vitro aspirin release from the PLLA films impregnated using the soaking method and the scCO_2_ impregnation method was carried out by immersing one film in 10 mL of 1 M PBS at 37 ± 1 °C. At predetermined intervals, 2 mL of the PBS solution were withdrawn and replaced with 2 mL of fresh PBS. The collected PBS samples were analyzed in a UV-Vis spectrometer (model UV-3600, Shimadzu, São Paulo, Brazil) at 298 nm for the aspirin quantification. The calibration curve was performed, and the molar absorptivity was found to be 0.3292 m^2^mol^−1^ at 298 nm. The release experiment was performed in triplicate and the cumulative release was calculated using Equation (5), considering that the 100% release is the release of the total amount of aspirin impregnated in each sample:

Cumulative release % = m_t_/m_impregnated_ × 100 (5)

where m_t_ is the total mass of the compound released at the time t and m_impregnated_ is the total aspirin mass impregnated in the film. The release kinetics and mechanism were evaluated, considering the possibility of a burst effect, using the Korsmeyer–Peppas model that can be applied to data up to 60% of the total release following the Equation (6) [52,53]:

M_t_/M = k.t^n^ + b (6)

where M_t_ is the released mass at the time t, M is the mass released at infinite time (which corresponds to m_impregnated_), k is a kinetic constant, b is the effect of a burst release, and n is the diffusional coefficient, which corresponds to a specific release mechanism as described in Table 1 [52].

### 2.11. Dissolution of the Aspirin Coating

The soaking-PLLA sample was covered with crystals of aspirin. The time necessary to dissolve the aspirin coating t_coating_ was determined using SEM images of soaking-PLLA films, that were immersed in 1 M PBS at 37 °C for different times. After the immersion in PBS, the samples were dried in a vacuum oven at 30 °C for 24 h and sputtered with a 15 nm layer of gold.

## 3. Results

### 3.1. Solvent Absorption

The polymer swelling directly affects the drug loading into the matrix. Thus, besides solubilizing aspirin, the solvent was selected to maximize the polymer swelling. PLLA absorbed more solvent than LLDPE for all solvents studied (Table 2). For both polymers, the scCO_2_ absorption was higher than the organic solvent absorption in the studied conditions, even though the contact time was shorter (3 h vs. 10 days).

### 3.2. Residual Solvent

Small amounts of residual solvent have been found in the soaking samples (Table 3) after 3 h of drying at 80 °C. For the samples treated with scCO_2_, the mass returned to its initial value 5 days after treatment, i.e., the complete desorption of CO_2_ was achieved and no residual solvent was present. Thus, all posterior characterizations of samples treated with scCO_2_ were performed after 5 days. The FTIR spectra of samples treated with organic solvents and scCO_2_ did not exhibit the characteristic peaks of the respective solvents (Supplementary Materials Section S1.2, Figure S1).

### 3.3. Drug Loading

**Figure 3:**
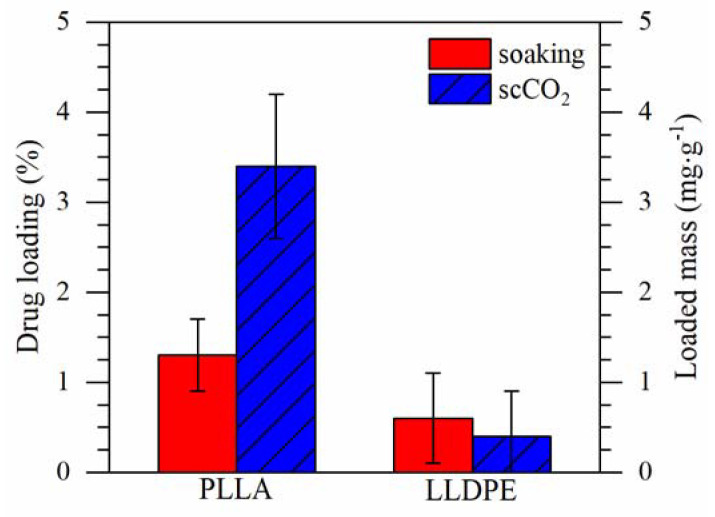
Drug loading (DL%) in approximately 40 mg of polymer.

The scCO_2_ impregnation resulted in a drug loading 2.6 times higher than the soaking method for PLLA (3.4 ± 0.8 vs. 1.3 ± 0.4%) (Figure 3). For LLDPE similar values were obtained with both methods (0.4 ± 0.5 vs. 0.6 ± 0.5%). The Tukey test revealed that only PLLA impregnated with the scCO_2_ method presented significant differences from the other samples, i.e., PLLA impregnated with soaking and both LLDPE had similar drug loadings.

Due to the low drug loadings observed for LLDPE, morphological, mechanical, in vitro degradation, and in vitro release assays were performed only for PLLA samples. Samples treated only with the solvents (Section 2.3) will be named ethanol-PLLA and scCO_2_-treated-PLLA and samples impregnated with aspirin will be named soaking-PLLA and scCO_2_-PLLA, for soaking and scCO_2_ impregnation, respectively.

### 3.4. Scanning Electron Microscopy (SEM)

**Figure 4:**
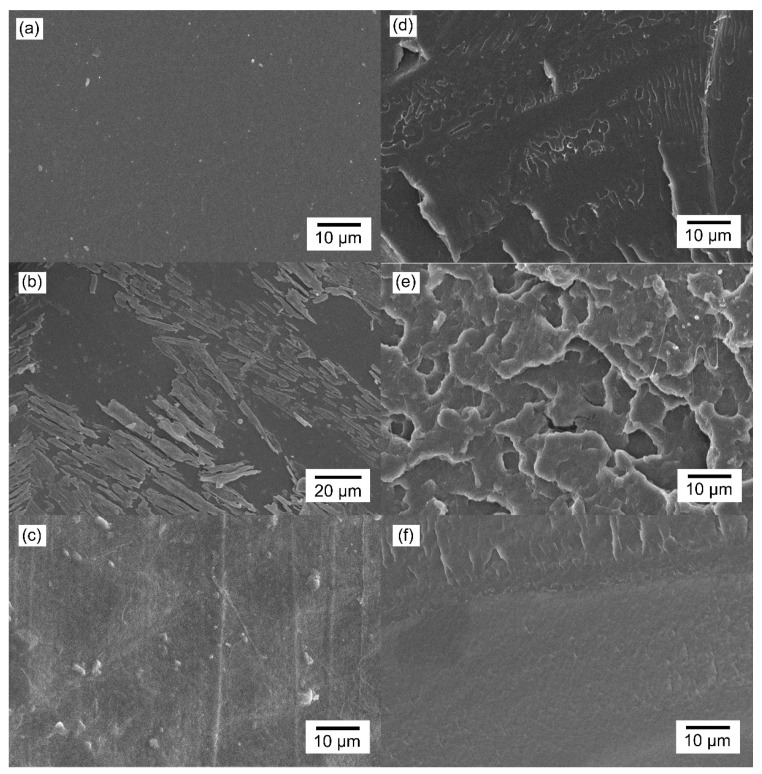
SEM images of (**a**) neat PLLA, (**b**) soaking-PLLA, and (**c**) scCO_2_-PLLA surfaces; and of (**d**) neat PLLA, (**e**) soaking-PLLA, and (**f**) scCO_2_-PLLA cross-sections.

SEM analyses were performed to observe the impact of the impregnation methods on the polymer surfaces and cross-sections. Needle-shaped crystals of aspirin with a width between 1 and 6 μm and a heterogeneous length between 6 and 120 μm were observed on the surface of the soaking-PLLA samples (Figure 4b). Large aggregates of parallel aspirin needles adhered to the PLLA surface but did not cover the entire surface (Supplementary Materials Section S3). However, such aspirin crystals were not observed on the surface of the scCO_2_-PLLA samples (Figure 4c) and the surface irregularities are attributed to the Teflon sheets in contact with the polymer used during hot pressing. The morphology of the neat PLLA cross-section was dense with no macropores (Figure 4d). Similarly, no macroporosity was evident in SEM images of the cross-section of scCO_2_-PLLA; despite the irregular fracture surface, the sample was dense and homogeneous (Figure 4f). The cross-section of the soaking-PLLA sample showed irregular polymer layers orientated along the cross-section, few irregular closed macropores were present, and the sample was less dense than neat PLLA (Figure 4e), as observed elsewhere for PLA in contact with ethanol and subsequent drying at 70 °C [54]. Supplementary Materials Section S3 presents additional images with lower magnifications (Figures S4–S6).

### 3.5. Thermal Analysis

**Figure 5:**
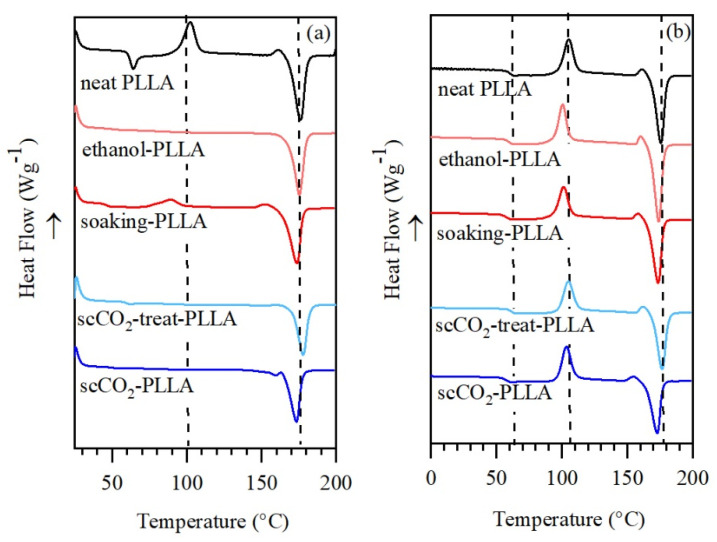
DSC thermograms of neat PLLA, PLLA treated with the impregnation solvent, and PLLA impregnated with aspirin using the soaking method and scCO_2_ method, (**a**) first heating and (**b**) second heating.

DSC analyses were performed to evaluate the effect of aspirin, ethanol, and scCO_2_ on the PLLA microstructure, and to investigate the chain mobility during the impregnation. In the first heating thermogram (Figure 5a), it is possible to evaluate the changes that occurred during the process. The neat PLLA thermogram exhibits a different glass transition temperature (T_g_) at 62.3 °C, a cold crystallization temperature peak (T_c_) at 102.2 °C, and it melts at 176 °C. The T_g_ of ethanol-PLLA, soaking-PLLA, and scCO_2_-treated-PLLA shifted to lower temperatures, whereas the T_g_ of scCO_2_-PLLA was not observed. The soaking-PLLA sample was the only one that exhibited a hot crystallization peak T_c_ (89.5 °C). PLLA crystallinity increased after the treatment with the pure solvents and after the two impregnation methods (Table 4). A negligible decrease of the melting temperature (T_m_) of approximately 3 °C was observed for both impregnated samples. In the second heating thermogram, the thermal history had been erased and the impact of the aspirin on PLLA can be observed (Figure 5b). T_g_ and the T_c_ were present in all thermograms and the characteristic temperatures were slightly shifted in comparison to PLLA (Table 4). No melting peak of aspirin was observed at 135 °C [55].

### 3.6. X-ray Diffraction

**Figure 6:**
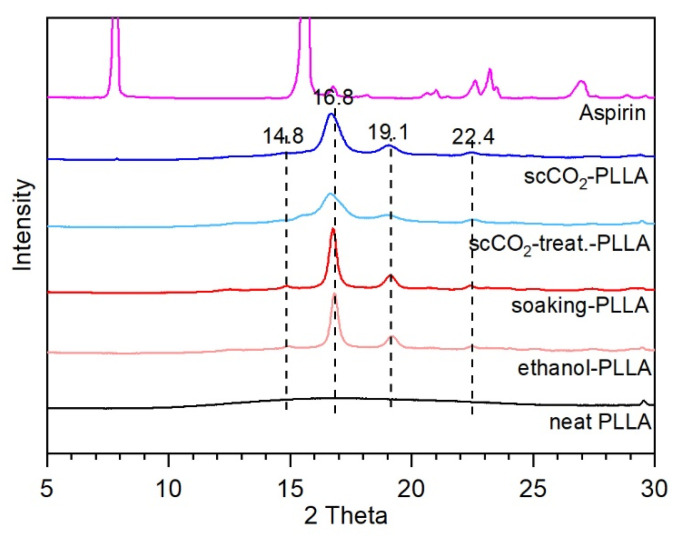
XRD patterns of neat PLLA, ethanol-PLLA, scCO_2_-treated-PLLA, soaking-PLLA, and scCO_2_-PLLA.

XRD analyses were performed to observe the changes in the PLLA crystallinity and the state of the loaded aspirin (Figure 6). Prior to the measurement, the surface of the samples was cleaned with wet tissue paper to remove any aspirin crystals on the surface, to only investigate the crystalline state of the aspirin loaded within the polymer. The XRD spectrum of the aspirin exhibits crystalline peaks, of which the most intense are centered at 7.8, 15.6, 23.2, and 27.0°. No characteristic peaks of aspirin were observed in the impregnated PLLA, which is in accordance with the DSC results. Although the DSC curves proved that neat PLLA is semicrystalline (Figure 5), the neat PLLA XRD spectrum exhibits a broad halo [20,56] in the studied range (the complete XRD pattern is presented in Supplementary Materials Section S4, Figure S7). Nevertheless, crystalline peaks appear on the spectra of the other samples (ethanol-PLLA, soaking-PLLA, scCO_2_-treated-PLLA, and scCO_2_-PLLA), at 14.8, 16.8, 19.1, and 22.4°. These peaks correspond to the formation of α-form PLLA crystals (orthorhombic unit cell with parameters of a = 1.06 nm, b = 0.61 nm, and c = 2.88 nm) [57,58] and correspond to the 010, 110/200, 100/203, and 102/210 plane reflections, respectively [54].

### 3.7. PLLA In Vitro Degradation

**Figure 7:**
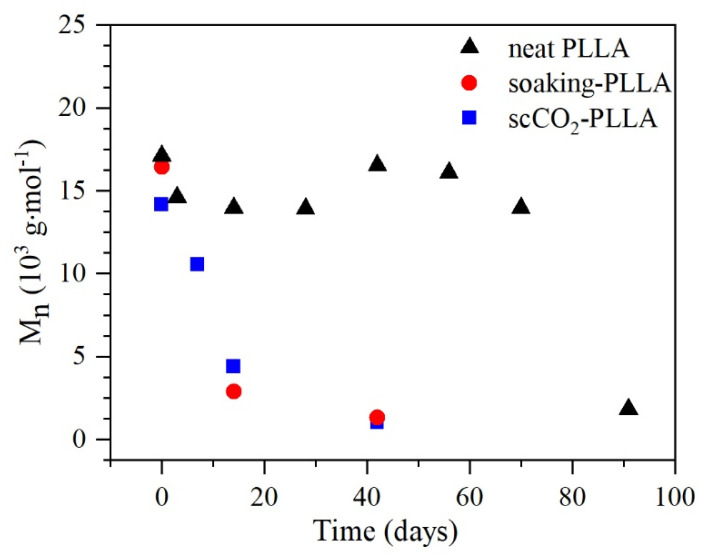
M_n_ of PLLA during the in vitro degradation experiment.

The M_n_ of the PLLA samples submitted to degradation over time (Figure 7) was estimated using 1H NMR analyses. The M_n_ of the initial samples (t = 0 days) is lower for the impregnated PLLA, showing that the impregnation process promoted some degradation and that it was higher for the scCO_2_-PLLA, which presented the lowest M_n_. Moreover, the M_n_ of neat PLLA decreases significantly only after 70 days, while the M_n_ of the impregnated samples decreased faster during the first 14 days, showing that the degradation is much faster for the impregnated samples. Finally, the degradation appears to be slightly faster for the soaking-PLLA sample.

**Figure 8:**
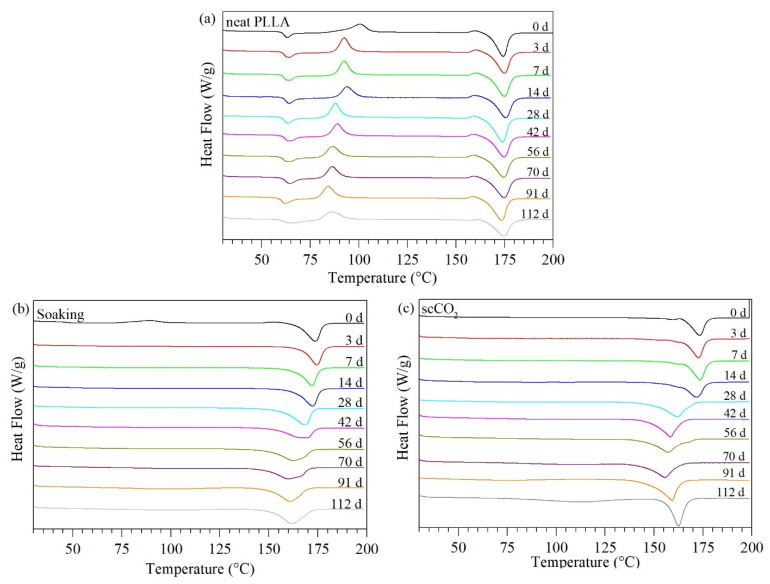
First heating DSC thermograms of PLLA during the degradation study at 37 °C in PBS of (**a**) neat PLLA, (**b**) PLLA impregnated using the soaking method, and (**c**) PLLA impregnated using scCO_2_ impregnation method.

**Figure 9:**
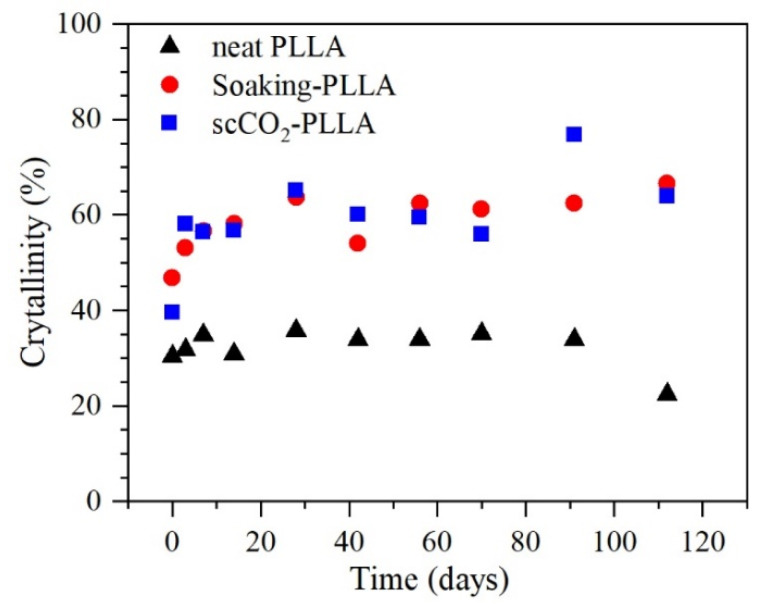
PLLA crystallinity evolution during the degradation study at 37 °C in PBS obtained with DSC analysis.

DSC analyses revealed the PLLA crystallinity and thermal properties evolution throughout the degradation study (Figure 8). For all samples, an increase in the crystallinity during the degradation study was observed (Figure 9). The neat PLLA crystallinity increased from 30.4% to 34.0% after 112 days (~3.7 months) in PBS, whereas the crystallinities of soaking-PLLA and scCO_2_-PLLA increased from 46.8% to 66.5% and from 39.6% to 63.9%, respectively, in the same period. The neat PLLA T_c_ decreased 8.2 °C over the degradation period while its T_g_ and T_m_ presented almost no change (<1 °C), according to Figure 8. The T_m_ of the samples impregnated using both techniques progressively shifted to lower temperatures, especially after 28 days of degradation.

### 3.8. In Vitro Aspirin Release

**Figure 10:**
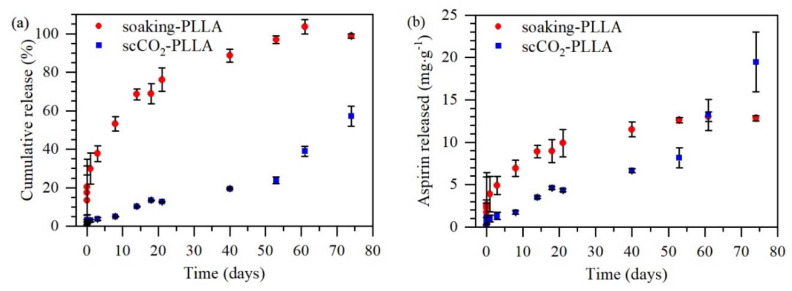
Comparison of the release profile of aspirin from soaking-PLLA and scCO_2_-PLLA samples, (**a**) cumulative release and (**b**) quantity of aspirin released.

**Figure 11:**
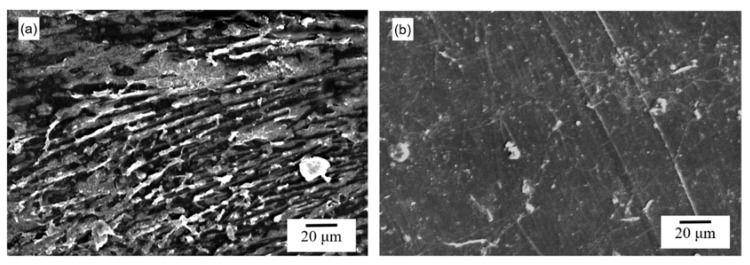
SEM images of soaking-PLLA immersed in 1 M PBS at 37 °C (**a**) for 30 min, and (**b**) for 1.5 h.

Aspirin release from soaking-PLLA and scCO_2_-PLLA samples was tested in PBS to verify whether the two methods could control drug release (Figure 10). The Korsmeyer–Peppas model that considers a possible burst effect, was applied to determine the release mechanism. Soaking-PLLA showed a burst release corresponding to c.a. 15% (release until 1.5 h) as indicated by parameter *b* in the Korsmeyer–Peppas model, related to Equation (6) (Table 5). The SEM images of the soaking-PLLA surface after immersion in PBS during different times showed that the aspirin crystals totally dissolved after 1 h 30, so t_coating_ was estimated to be 1.5 h (Figure 11). Conversely, scCO_2_-PLLA did not exhibit such a burst release, parameter *b* of the Korsmeyer–Peppas model being smaller than the deviation, and only 3% of impregnated aspirin was released within 24 h, which is consistent with the absence of aspirin on the surface (Figure 4c). The release of aspirin from scCO_2_-PLLA was much slower than from soaking-PLLA. After 74 days, c.a. 60% of the drug was released from scCO_2_-PLLA, whereas the whole aspirin content was released from soaking-PLLA after 60 days. According to the kinetic parameter *n* of the samples (Table 5), aspirin was released from soaking-PLLA according to the Fickian diffusion mechanism whereas its release from scCO_2_-PLLA occurred through diffusion and matrix erosion.

## 4. Discussion

### 4.1. Solvent Absorption

Solvent absorption occurs in the amorphous regions of the polymer and it depends on the crystallinity, the affinity between polymer and solvent, and the chain mobility of the amorphous regions [29,59]. According to DSC measurements, the crystallinity of neat PLLA and LLDPE are comparable (30.4 and 31.8%, respectively); thus, this cannot explain the difference in the initial solvent absorption. The solubility parameter (δ) (Table 6), derived using the Flory–Huggins equation, can predict the polymer/solvent affinity. The closer the solubility parameters of two organic compounds, the higher their affinity and the higher the expected absorptions [60]. As reported in Table 6, higher PLLA/ethanol affinity is predicted than LLDPE/isopropanol affinity, and higher solvent absorption was observed for the PLLA. The higher absorption of scCO_2_ in both polymeric matrices compared with the organic solvents can be attributed to the high diffusivity of supercritical fluids [25,61]. While PLLA has ester groups in its structure (Figure 1a) that interact with CO_2_ and ethanol [62,63,64], LLDPE has no polar groups (Figure 1b), explaining the higher solvent absorption of PLLA regardless of the solvent.

In addition, the absorbed solvent has a plasticizing effect, as indicated by the decrease in PLLA T_g_ (Figure 5), increasing the chains’ mobility in the amorphous regions. The increased mobility can result in two competing effects: it can promote solvent absorption and allow the polymeric chains to organize into crystalline regions, reducing the chain mobility progressively and, consequently, decreasing the solvent absorption [54,59,65]. In the conditions used for scCO_2_ impregnation, PLLA was above its T_g_ (62 °C); thus, the amorphous phase had more mobility in the scCO_2_ method than in the soaking method and more solvent was absorbed. The fact that LLDPE was above its T_g_ (−110 °C) in both methods but did not absorb high quantities of solvent confirms that its low affinity with the solvents was the main parameter governing the solvent absorption in this polymer, as shown in Table 2. In a previous study, we reported that the mass of CO_2_ sorbed in LLDPE is less than half that sorbed in PLLA in the same conditions (6.1 ± 2.6 wt.% vs. 14.2 ± 3.2 wt.%) [47].

### 4.2. Residual Solvent

The volatile nature of the solvents can explain the small residual solvent values. Moreover, for the organic solvents, the residual solvent values followed their affinities with the polymeric matrices (Table 3). FDA standards classify ethanol and isopropanol as solvents of Class 3, which corresponds to less toxic solvents and a lower risk to human health, requiring a maximum of 50 mg of ethanol or isopropanol released in the organism per day; while no information was found about CO_2_ [66]. For each 40 mg of the sample impregnated through soaking, approximately 0.36 mg of the residual solvent remained trapped (Table 3), which is within the regulations for pharmaceutical applications. However, the amount of residual solvent is proportional to the sample dimensions; thus, when working with larger samples, it will be necessary to verify if the amount of residual solvent meets the required standards. If not, the solvent removal process must be adapted, using vacuum drying, for example.

### 4.3. Drug Loading

The higher drug loading obtained using the scCO_2_ impregnation method can be explained by the higher scCO_2_ absorption than the absorption of organic solvents in the matrixes, which, as discussed, results in higher polymer swelling and facilitates the drug diffusion in the matrix. Thus, even though aspirin is more soluble in organic solvents (Table 7), more aspirin molecules are carried into the polymers by scCO_2_.

As already discussed, the closer the solubility parameters of two organic compounds, the higher their affinity. Good polymer/drug affinity, promoted by interactions such as Van der Waals and/or H-bonding, is known to enhance the drug loading by drug retention in the matrix rather than in the solvent phase [46,67,68,69]. Thus, besides absorbing more solvent, the higher drug loading of PLLA can be explained by the higher PLLA/aspirin affinity than LLDPE/aspirin affinity, as demonstrated with the solubility parameter (Table 6).

The scCO_2_ impregnation method not only enables higher drug loadings, but there is also a shorter processing time than the soaking method. The soaking was carried out for 10 days (Supplementary Materials Section S2, Figures S2 and S3), whereas scCO_2_ impregnation lasted 3 h. In addition, the soaking method required three additional hours to remove the solvents, whereas the scCO_2_ impregnation led to samples free of residual solvent 5 days after the impregnation without additional steps. Other works have reported higher drug loadings using scCO_2_ impregnation than the soaking method [21,36,37]. Costa et al. [36] observed that the amount of flurbiprofen loaded in a Methafilcon A contact lens was seven times higher using the scCO_2_ impregnation method (40 °C, 9 MPa, 2 h) than that obtained using the soaking method (PBS, 48 h).

Furthermore, the drug loading is easily tunable by controlling the scCO_2_ impregnation conditions, i.e., the temperature, pressure, and depressurization rate [33,46,72]. Aspirin drug loading has been shown to vary between 0 to 72.1% when varying the impregnation conditions between 35 and 140 °C and 7.5 and 35 MPa, and depending on the polymer, including PLLA, poly(d,l-lactic acid) (P(d,l)LA), polyethylene terephthalate (PET), polypropylene (PP), and β-glucan aerogels [46,73,74]. The higher drug loading was obtained in P(d,l)LA at 40 °C and 8.0 MPa. For LLDPE, the drug loadings achieved by scCO_2_ impregnation range from 0.34 to 6.0% [75]. The lower drug loadings in LLDPE than in PLLA are attributed to its lower CO_2_ absorption [47].

Although the loaded drug can recrystallize into the matrix [76] the absence of aspirin diffraction peaks in the XRD patterns and aspirin melting peak in the DSC thermograms indicate that the impregnated aspirin was in the amorphous state in the polymer bulk as a reflection of its affinity with PLLA [72,76]. The crystalline state of the loaded drug directly impacts its release since crystalline forms are known to solubilize more slowly than the amorphous forms, thus presenting a slower release rate [77,78,79].

Finally, aspirin did not present any degradation or degradation products after scCO_2_ impregnation (Supplementary Materials Section S5, Figure S8), even though this process required higher pressure and temperature.

### 4.4. PLLA Crystallinity

The increase in PLLA crystallinity observed in both methods can be attributed to the plasticizing effect of the solvents because higher crystallinities were achieved when the PLLA samples were treated in the absence of aspirin. As previously discussed, solvent absorption in the amorphous phase increases the polymeric chain mobility, allowing the chains to reorganize into crystals [54,59,65]. Moreover, the drying step performed to remove ethanol after the soaking was performed at 80 °C, which is in the cold crystallization region of soaking-PLLA (T_c_ = 89.5 °C). Therefore, the crystallinity increased during the soaking and the drying step, achieving the highest value among the samples (54.1%), consistent with the intense diffraction peaks (Figure 6). The small changes in the melting peak shape on DSC traces indicate that the crystals formed during the impregnation process are very similar to the existing ones. For each method, the DSC and XRD patterns of PLLA only treated with solvent and impregnated with aspirin are similar. Thus, aspirin does not significantly impact PLLA morphology when impregnated in such a drug loading range.

### 4.5. PLLA In Vitro Degradation

PLLA degrades in humid environments and in vivo by hydrolysis of its ester bonds (Figure 1a), resulting in a reduction of the molecular weight and changes in morphology and mechanical and thermal properties [31,58,59].

NMR results (Figure 7) showed the reduction of PLLA M_n_ prior to the degradation study and the fast degradation of the impregnated samples compared to neat PLLA. Acid groups in the drug structure, such as the carboxyl group of aspirin (Figure 1c), are known to increase the degradation rate [31,80] and, thus, can explain the observed results. The highest reduction of scCO_2_-PLLA M_n_ prior to the degradation assay can be explained by its higher impregnation temperature and drug loading.

Crystalline regions in a semicrystalline polymer such as PLLA are characterized as being organized and packed more tightly. Therefore, the diffusion of water molecules within the polymer occurs first in the amorphous regions, and hydrolysis occurs preferentially in the amorphous segments. The breakage of the polymeric chains increases their mobility, allowing them to reorganize into a more ordered structure, which explains the increase in crystallinity (Figure 9) [58,81]. This increased mobility is also evidenced by the decrease in the T_g_ on the second heating curve (Supplementary Materials Section S6, Figure S9) and in the decrease in the T_c_ of neat PLLA (Figure 8) that shows a facilitated crystallization [58,82].

When the degradation continues for longer periods, the crystalline regions start to degrade, resulting in a decrease in the crystals’ thickness and, consequently, lower energy required to melt them [81,83]. The formation of smaller crystals is evidenced by the decrease of the T_m_ of the impregnated samples after 28 days (Figure 8b,c). Such a shift was not observed in the neat PLLA (Figure 8a), showing that the presence of aspirin indeed accelerates the degradation process.

The degradation process must match the healing speed of the tissue in which the polymer is implanted: if it is faster, the polymer loses its structural function, and if it is too slow, the tissue regeneration is hindered [84,85]. It was shown that the use of a drug with acid groups promotes fast degradation of PLLA, and, therefore, the impact of the choice of drug on the degradation rate must be considered. Knowing that PLLA has long degradation times, approximately 90 days, that often do not match those of the tissue healing process [86,87], the impregnation of drugs bearing acid groups may help to accelerate PLLA degradation. On the other hand, if the degradation rate of PLLA must be maintained, aspirin should be substituted with a drug without acid groups.

### 4.6. In Vitro Release

Soaking-PLLA exhibits a burst release of c.a. 15% of its drug loading according to the Korsmeyer–Peppas model. This burst release can be attributed to the dissolution of the needle-shaped crystals of aspirin present on the surface (Figure 4b). These crystals are a result of the fast decrease in aspirin solubility when the sample is removed from the solution and the residual ethanol on the surface evaporates. The aspirin crystals on soaking-PLLA dissolved after 1.5 h. Two polymorphs of aspirin exist: form I is a stable form, while form II is metastable [55,88]. Mittal et al. [88] observed such needle-shaped morphology for crystals identified as form I, according to DSC, FTIR, and XRD characterizations. Consequently, the crystals observed on soaking-PLLA may be attributed to the stable form I of aspirin, which has a slower dissolution rate than form II.

For scCO_2_ impregnation, aspirin did not precipitate on PLLA during the fast cooling (−78 °C) performed before depressurization (Figure 4c). In contrast, such a phenomenon was previously observed for ketoprofen in the same conditions [31], highlighting that the formation of a coating of drugs on the polymer may depend on the drug.

The aspirin release mechanism of soaking-PLLA was governed by Fickian diffusion and the aspirin release mechanism of scCO_2_-PLLA was driven by diffusion and polymer erosion. The slight difference in the degradation rate of the two matrices does not account for such distinct release profiles and mechanisms. The quicker aspirin release from soaking-PLLA may be related to a limitation of the soaking method that generally results in drug impregnation closer to the surface of the polymer [16,24]. The irregular intern morphology and presence of few macropores in this sample, a sparser morphology formed during soaking time as a result of the solvent absorption, and solvent-induced crystallization [54] (Figure 4e) may favor the diffusion of the drug up to the release medium. On the contrary, the scCO_2_-impregnation method enabled loading aspirin into the bulk of PLLA due to the good scCO_2_ diffusivity, and a dense matrix was obtained [25,61]. According to the modelization of the release profile, the progressive scCO_2_-PLLA degradation contributes to control aspirin release. Thus, prolonged aspirin release was achieved and 19.5 μg∙g^−1^ of aspirin was released during 74 days.

## 5. Conclusions

This paper investigated aspirin loading in PLLA and LLDPE through soaking and supercritical CO_2_ (scCO_2_) impregnation methods to develop local delivery systems as an alternative to oral administration. The scCO_2_ impregnation method was a faster process, promoted higher drug loadings in PLLA, and resulted in systems free of solvent residues. Higher drug loadings were obtained in PLLA samples due to their higher solvent absorption and affinity with aspirin, in addition, needle-shaped aspirin crystals were observed on the PLLA sample impregnated by soaking. Both methods increased the PLLA crystallinity and resulted in amorphous aspirin in the matrix, evidenced by the absence of aspirin T_m_ and crystalline peaks of aspirin in DSC and XRD curves, respectively. The in vitro degradation of PLLA samples from both methods was similar, showing a faster degradation rate than neat PLLA as an effect of the aspirin acid groups that catalyze PLLA hydrolysis. Finally, aspirin in vitro release from soaking-PLLA was faster and governed by Fickian diffusion with a burst release of 15% in the first 1.5 h, whereas the release from scCO_2_-PLLA was slower, with 3% released in the first 24 h, and depended not only on aspirin diffusion but also matrix degradation leading to c.a. 60% of aspirin released after 74 days vs. 100% after 60 days for soaking-PLLA. This prolonged release from scCO_2_-PLLA could be a substitution for oral long-term aspirin administration, which may cause gastrointestinal adverse events. The scCO_2_ impregnation method presented a good alternative to the conventional soaking, due to its higher drug loading, similar degradation profile, and more sustained release.

## Figures and Tables

**Table 1:** Release mechanism interpretation of the diffusional coefficient of the Korsmeyer–Peppas model for a polymeric film [52].

Diffusional Coefficient (*n*)	Release Mechanism
≤0.5	Fickian diffusion
0.5 < *n* < 1.0	Anomalous transport—diffusion and matrix erosion
1.0	Case-II transport
>1.0	Super Case-II transport

**Table 2:** Solvent absorption measured after 10 days of immersion at room temperature in the case of ethanol and isopropanol solvents, and at 80 °C, 30 MPa, and after 3 h for scCO_2_.

Polymer	Solvent	Solvent Absorption (%)
PLLA	ethanol	6.0 ± 0.8
	scCO_2_	14.2 ± 3.2
LLDPE	isopropanol	0.4 ± 0.3
	scCO_2_	6.1 ± 2.6

**Table 3:** Residual Solvent.

Polymer	Solvent	Residual Solvent (%)
PLLA	Ethanol	0.9 ± 0.4% (0.90 ± 0.09% ^1^)
	scCO_2_	0.0 ± 0.0%
LLDPE	Isopropanol	0.5 ± 0.5%
	scCO_2_	0.0 ± 0.0%

^1^ Determined by ^1^H NMR.

**Table 4:** Crystallinity and thermal transitions of neat PLLA, PLLA treated with the impregnation solvent, PLLA impregnated with aspirin by soaking method and by scCO_2_ method.

Sample	First Heating				Second Heating			
	T_g_ (°C)	T_c_ (°C)	T_m_ (°C)	χ (%)	T_g_ (°C)	T_c_ (°C)	T_m_ (°C)	χ (%)
Neat-PLLA	62.3	102.2	176.0	30.4	60.2	105.5	175.7	28.3
Ethanol-PLLA	51.0	-	175.4	54.1	56.9	100.6	174.1	33.8
Soaking-PLLA	46.9	89.5	173.7	46.8	58.2	101.4	173.6	29.1
scCO_2_-treated PLLA	59.1	-	177.8	42.4	62.4	105.1	176.6	29.3
scCO_2_-PLLA	-	-	173.4	39.6	57.8	103.6	172.9	21.2

**Table 5:** Release kinetic parameters obtained from the Korsmeyer–Peppas model for soaking-PLLA and scCO_2_-PLLA.

	n	b	k	R^2^
soaking-PLLA	0.50 ± 0.05	15.10 ± 2.01	13.33 ± 2.15	0.995
scCO_2_-PLLA	0.76 ± 0.13	1.32 ± 1.51	1.14 ± 0.61	0.930

**Table 6:** Solubility parameters calculated using the Hoftyzer–Van Krevelen method [60].

Compound	Solubility Parameter (δ) (MPa^0.5^)	|δ_PLLA_—δ_solvent/aspirin_| (MPa^0.5^)	|δ_LLDPE_—δ_solvent/aspirin_| (MPa^0.5^)
PLLA	23.3	-	-
PE	17.6	-	-
Ethanol	25.6	2.3	8.0
Isopropanol	22.9	0.4	5.3
Aspirin	25.7	2.4	8.1

**Table 7:** Aspirin solubility in ethanol and isopropanol at 18 °C and in scCO_2_ at {80 °C; 30 MPa}.

Solvent	Aspirin Solubility (M Fraction)	Reference
Ethanol	4.9 × 10^−2^	[70]
Isopropanol	3.2 × 10^−2^	[70]
scCO_2_	9.2 × 10^−4^	[71]

## Data Availability

Not applicable.

## Supplementary Materials

The following is available online at https://www.mdpi.com/article/10.3390/pharmaceutics13060824/s1, Supplementary Materials containing Figure S1: FTIR spectra of polymer samples after solvent removal compared to the neat polymers and pure solvents (a) PLLA samples and (b) LLDPE samples; Figure S2: Swelling of PLLA in ethanol and LLDPE in isopropanol; Figure S3: Swelling in aspirin solution in ethanol for PLLA and isopropanol for LLDPE; Figure S4: Neat PLLA (a) surface and (b) cross-section; Figure S5: Soaking-PLLA (a) surface and (b) cross-section; Figure S6: scCO2-PLLA (a) surface; Figure S7: XRD pattern of neat PLLA; Figure S8: 1H NMR spectra of aspirin after solubilization in scCO2 at 140 °C and 30 MPa; Figure S9: DSC 2nd heat thermograms of PLLA during the degradation study (a) neat PLLA, (b) PLLA impregnated through the soaking method and (c) PLLA impregnated through the scCO2 impregnation method.
