# Supplementary material for: Aspirin-Loaded Polymeric Films for Drug Delivery Systems: Comparison between Soaking and Supercritical CO2 Impregnation
Source: Pharmaceutics. 2021 Jun 2;13(6):824. doi: 10.3390/pharmaceutics13060824 (PMC8229088; doi:10.3390/pharmaceutics13060824)
Supplement: Supplementary file 1 [file pharmaceutics-13-00824-s001.zip › pharmaceutics-1230469-SI.pdf]

pharmaceutics

MDPI

Supplementary Materials: Aspirin-Loaded Polymeric Films for Drug Delivery Systems: Comparison between Soaking and Supercritical CO_2 Impregnation

Isabela Trindade Coutinho, Lígia Passos Maia-Obi and Mathilde Champeau

S1. Residual Solvent

S1.1.^{1}H NMR

Proton nuclear magnetic resonance (^{1}H NMR) analysis was performed to evaluate the content of ethanol in the soaking-PLLA sample. Approximately 10 mg of sample was dissolved in 0.5 ml of deuterated chloroform ($CDCl_3$) and the spectrum was recorded in a Varian VNMRS 500 MHz spectrometer at 27 °C, using 800 transients. The content of ethanol was determined from the comparison of the signal integration of the methylene group of the ethanol (δ = 3.72 ppm) and the PLLA methine ester end group (δ= 4.36 ppm) and the PLLA methine ester groups (δ = 5.20 ppm). The error in the determination is estimated to be lower than 10 %.

S1.2. FTIR

In the range of 3650 to 2270 cm^{-1} of the FTIR spectra (Figure S1), ethanol possesses two characteristic peaks, the υ_{O-H} stretching at 3332 cm^{-1} and the υ_{C-H} peak at 2976 cm^{-1}. In the same range, isopropanol exhibits peaks corresponding to υO-H stretching and the υ_{C-H} at 3336 and 2970 cm^{-1}, respectively. Finally, gaseous CO_2 has the $2_{\upsilon 2+\upsilon 3}$ peak close to 3600 cm^{-1} and the $\upsilon_3{}^{13c}$ peak centered at 2283 cm^{-1}. No solvent peaks were observed in both PLLA spectra. For LLDPE, a small peak at 3336 cm^{-1} can be observed in the soaking samples, which could be attributed both to of isopropanol υ_{O-H} stretching or to water presence. Thus, it is not possible to conclude the presence of residual solvent from this analysis.

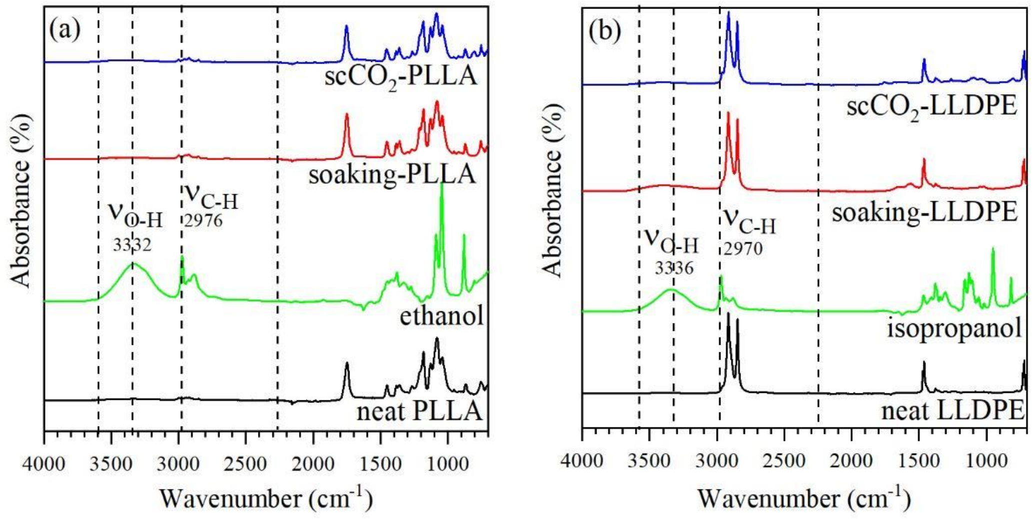

Figure S1. FTIR spectra of polymer samples after solvent removal compared to the neat polymers and pure solvents (**a**) PLLA samples and (**b**) LLDPE samples.

S2. Swelling Evolution in the Soaking Method

PLLA had an increase in its swelling from day 0 to day 6, whereas LLDPE showed no increase after 2 days. Therefore, 10 days of soaking was chosen as a standard to ensure equilibrium and maximal solvent absorption.

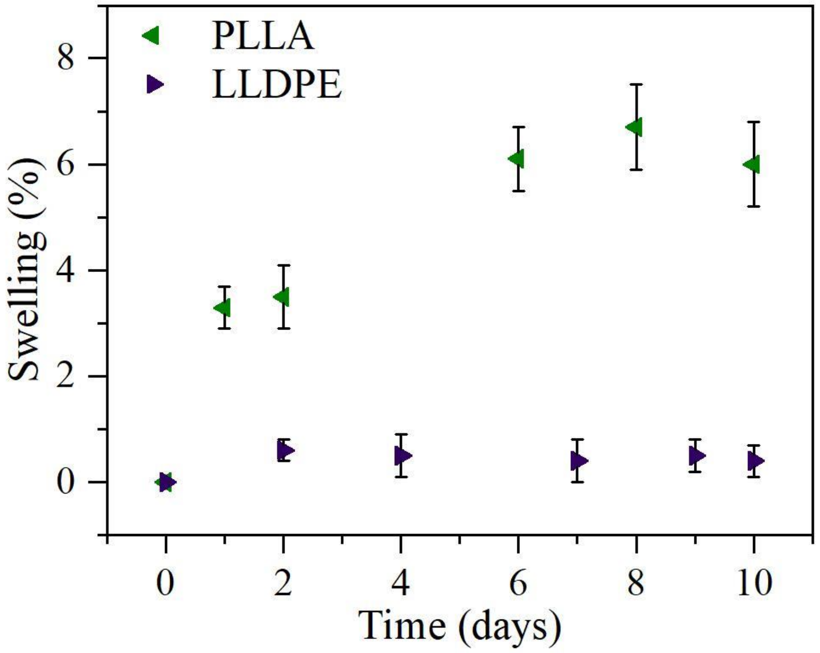

Figure S2. Swelling of PLLA in ethanol and LLDPE in isopropanol.

When the polymers were immersed in the aspirin solution, the swelling profile was similar to the pure solvent. PLLA swelling increased up to 6 days and LLDPE swelling did not increase after 2 days of soaking.

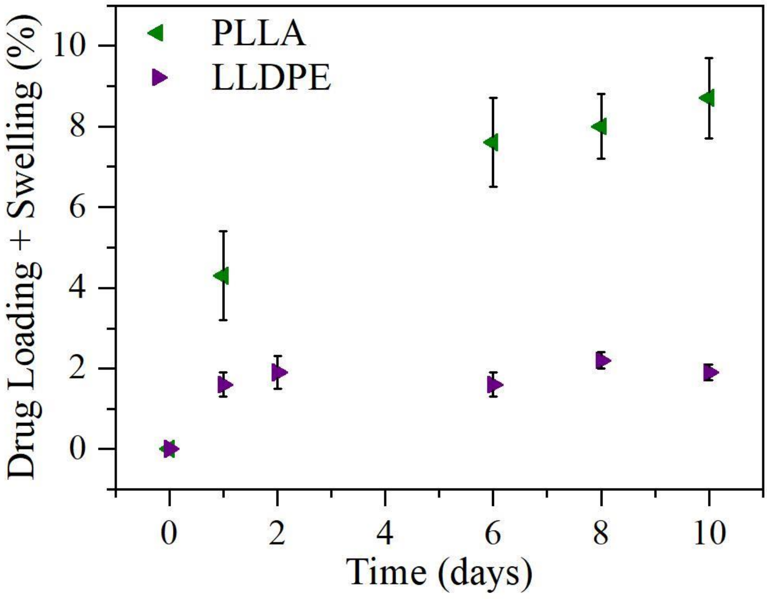

Figure S3. Swelling in aspirin solution in ethanol for PLLA and isopropanol for LLDPE.

S3. Additional SEM Images with Lower Magnifications

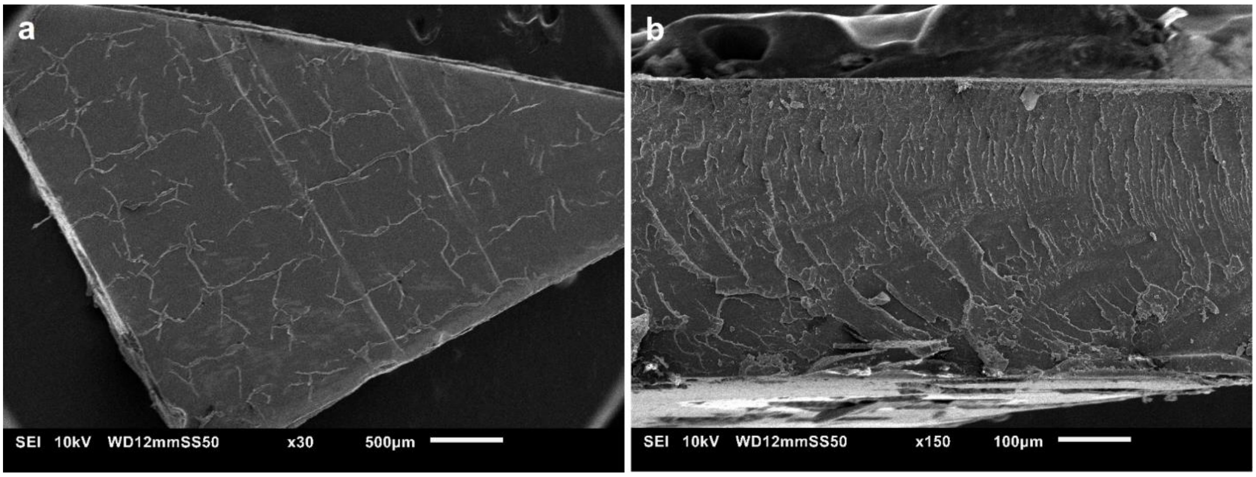

Figure S4. Neat PLLA (**a**) surface and (**b**) cross section.

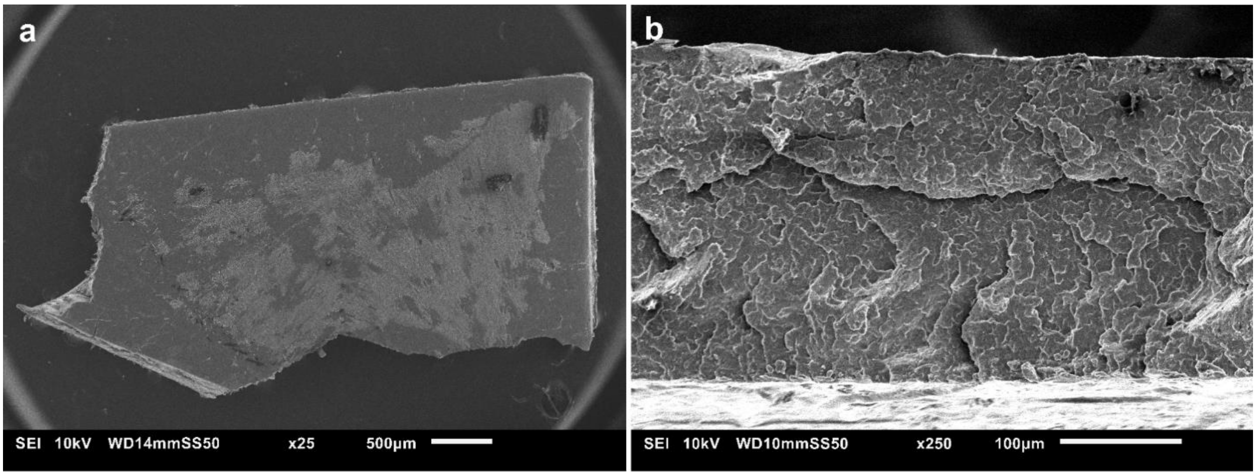

Figure S5. Soaking-PLLA (**a**) surface and (**b**) cross section.

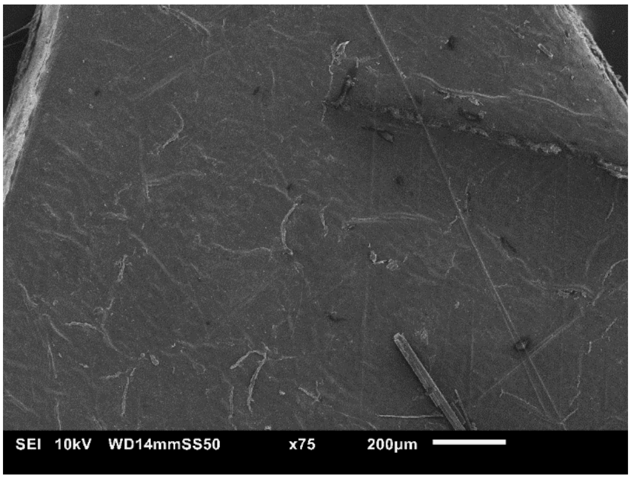

Figure S6. ScCO_2-PLLA surface.

S4. Neat PLLA XRD Pattern

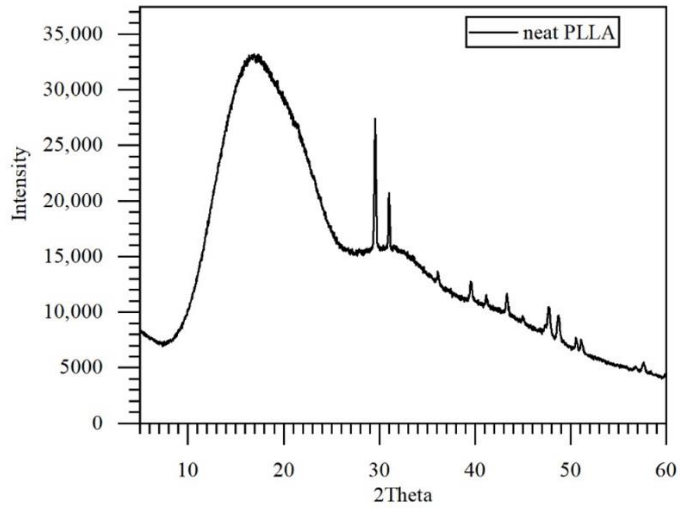

Figure S7. XRD pattern of neat PLLA.

S5. Evaluation of Aspirin After the Treatment With $scCO_2$

Aspirin was processed in $scCO_2$ with a protocol similar as described in section *2.4.2 $scCO_2$ impregnation method*, but without the addition of polymers into the reactor and using conditions more extreme to ensure that no aspirin degradation would be observed during the $scCO_2$ impregnation process. Thus, the structure of the aspirin precipitated in the reactor after being treated in $scCO_2$ at 140 C and 30 MPa for 5h was analyzed by ^1H NMR, using $CDCl_3$ as solvent. The aspirin NMR spectrum is presented in Figure S7. The sample does not present impurities significantly and no acetic acid (δCH_3= 2.10 ppm), a product of decomposition of aspirin, was identified. Consequently, it can be concluded that aspirin does not undergo degradation during $scCO_2$ processing.

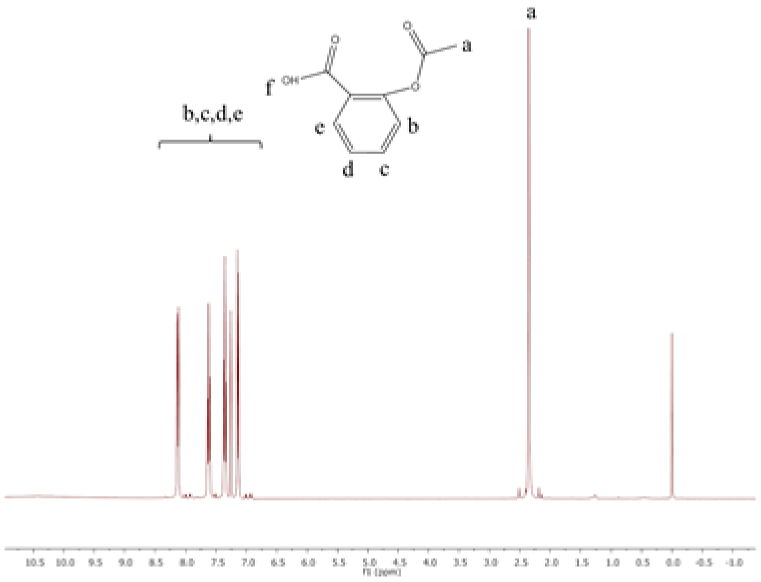

Figure S8. 1H NMR spectra of aspirin after solubilization in $scCO_2$ at 140 °C and 30 MPa.

S6. Degradation Evolution—DSC Data

Figure S8 shows DSC 2nd heat thermograms of PLLA during the degradation study. The glass transition temperature (T_g) is decreasing throughout the degradation study, evidencing the increase in the mobility in polymeric chains.

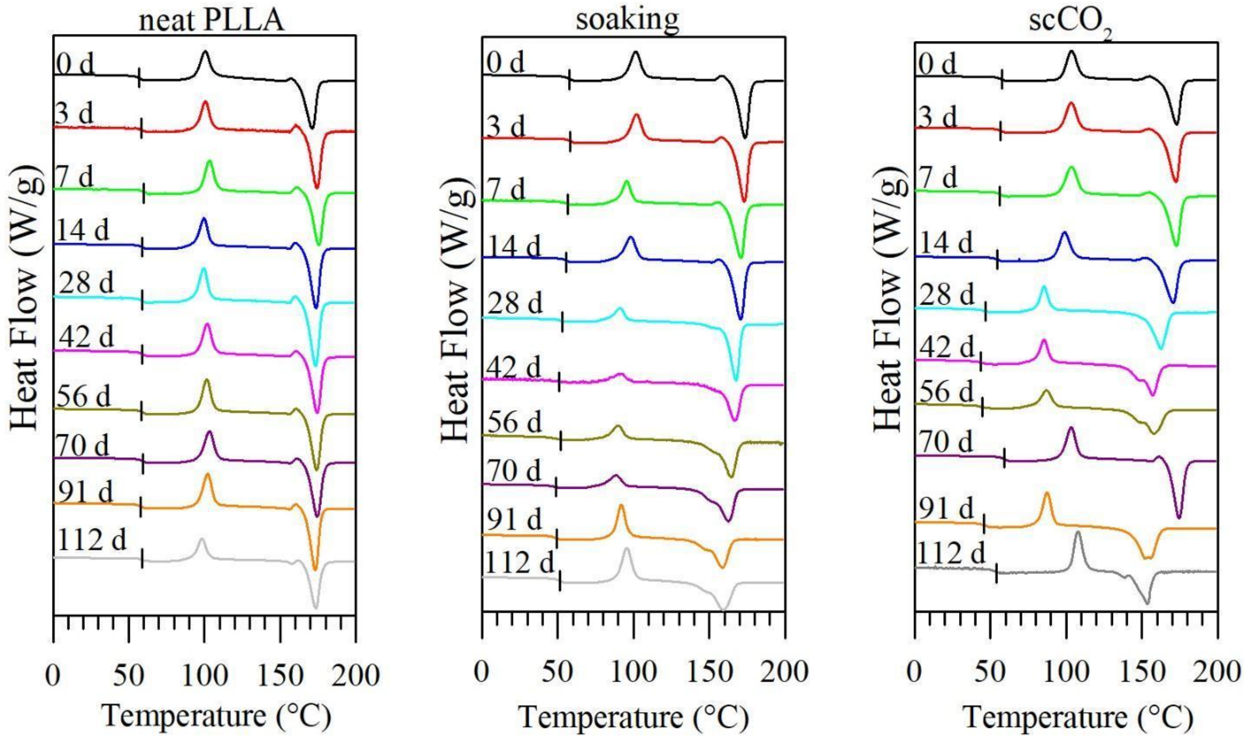

Figure S9. DSC 2nd heat thermograms of PLLA during the degradation study (**a**) neat PLLA, (**b**) PLLA impregnated through the soaking method and (**c**) PLLA impregnated through the $scCO_2$ impregnation method.
